# Pregnancy in Women With Klippel–Trenaunay Syndrome Complicated by Uterine Vascular Malformation: A Case Report and Literature Review

**DOI:** 10.7759/cureus.90065

**Published:** 2025-08-14

**Authors:** Ruijing Chang, Jingyu Zhang, Feng Zhang, Lina Wen, Yuping Jiang

**Affiliations:** 1 Obstetrics, The Second Hospital of Hebei Medical University, Shijiazhuang, CHN; 2 Haematology, The Second Hospital of Hebei Medical University, Shijiazhuang, CHN; 3 Vascular Surgery, The Second Hospital of Hebei Medical University, Shijiazhuang, CHN

**Keywords:** congenital vascular disorder, klippel-trenaunay syndrome, multidisciplinary collaboration, pregnancy, uterine involvement

## Abstract

Klippel-Trenaunay syndrome (KTS) is a rare congenital vascular disorder characterized by the classical triad of cutaneous port-wine stain (capillary hemangioma), varicose veins, and hypertrophy of soft tissues and bones. KTS with uterine involvement is extremely uncommon. We present a 27-year-old pregnant woman with KTS complicated by uterine venous malformation. Comprehensive management was provided by a multidisciplinary team after 12 weeks gestation diagnosed with KTS. The patient wore full-length compression stockings and received low-molecular-weight heparin (LMWH) for thromboprophylaxis. At 34 weeks gestation, imaging studies, including colour Doppler ultrasound and pelvic MRI, were performed to evaluate venous malformations throughout the myometrium. Given varicose veins affecting the lumbar region and vagina, an elective cesarean section under general anesthesia was performed at 37 weeks. The patient’s total blood loss of 2200 mL. An infant weighing 2,940 g was delivered with Apgar scores of 8/9/10. Postoperatively, prophylactic LMWH 5000 IU once daily was resumed, and based on marked elevations in fibrin degradation products (FDP), plasmin-antiplasmin complex (PIC), D-dimer, and thrombin-antithrombin (TAT), along with a decline in fibrinogen, adjusted LMWH 5000 IU twice daily for six weeks. This case highlights the challenges of managing pregnancy in women with KTS complicated by uterine venous malformation.

## Introduction

Klippel-Trènaunay syndrome (KTS) is a rare congenital vascular disorder first described in 1900 by French physicians, Klippel and Trènaunay, as a syndrome characterized by osteohypertrophic varicose nevus [1]. The estimated prevalence of KTS ranges from one in 30,000 to 100,000 live births, with a male-to-female ratio of 9:10, and it typically occurs sporadically without a predilection for sex or race [2]. Recent research suggests that KTS may be associated with mutations in the PIK3CA gene [3]. Genetic studies link sporadic chromosomal translocations (t[5;11], t[8;14]) to somatic mutations during embryogenesis that dysregulate angiogenesis, notably overexpression of the vascular factor VG5Q (AGGF1) on chromosome 5 [4]. KTS primarily affecting the visceral involvement is rare but has been documented in the colon, small bowel, bladder, kidney, spleen, liver, mediastinum and brain. Pregnant individuals with KTS may experience recurrent thromboembolism and severe postpartum hemorrhage because of increased progesterone levels, hypercoagulability, elevated circulating blood volume, and high cardiac output in pregnancy. Our case highlights the meticulous management of uterine involvement in KTS, which includes preconception and postpartum anticoagulation, dynamic monitoring of D-dimer and thrombin-antithrombin III complex (TAT) levels, and delineated critical coagulation transitions, markedly reducing serious adverse events.

## Case presentation

**Figure 1:**
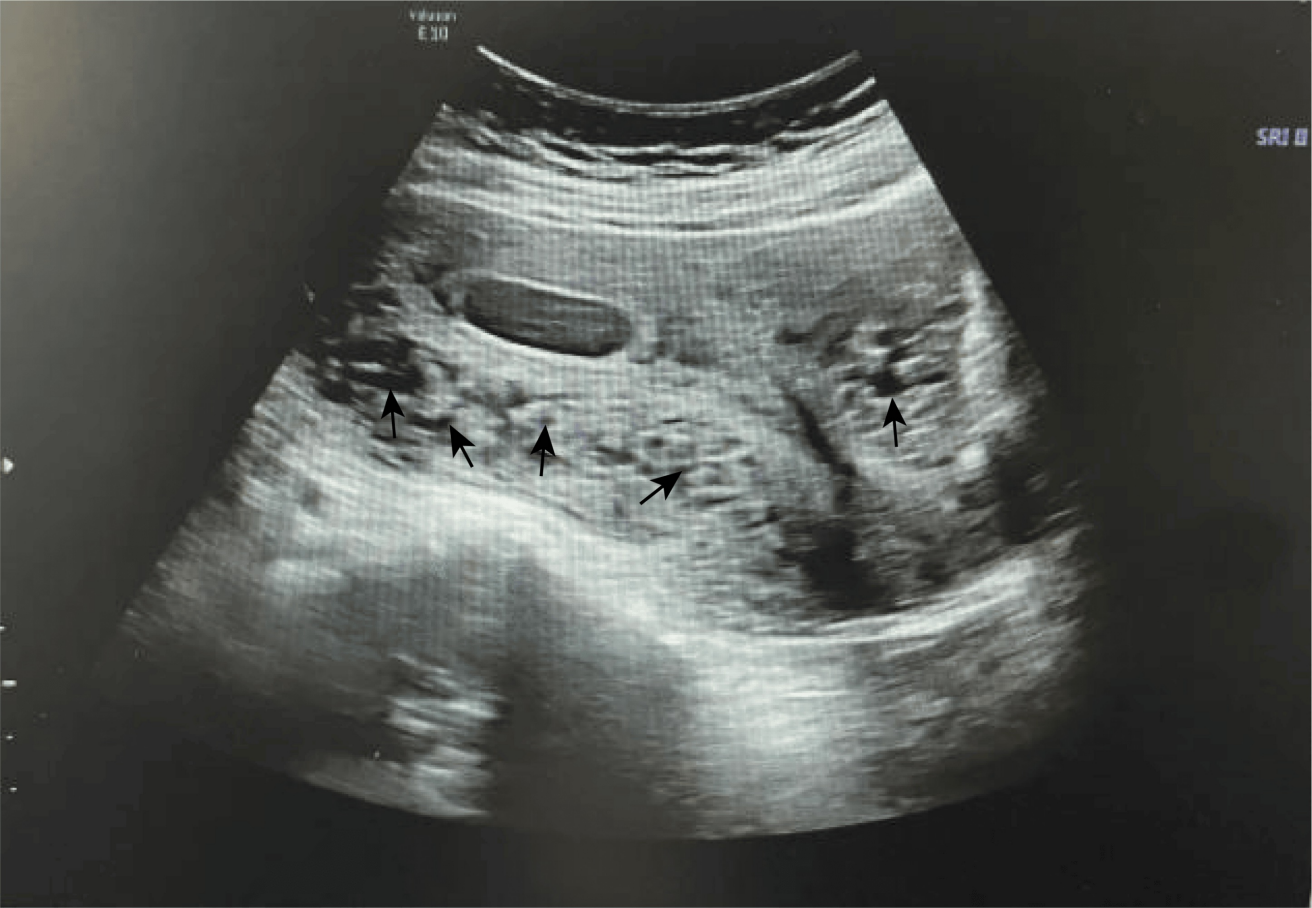
At the onset of pregnancy, the affected uterine wall exhibits an initial enlargement of the luminal diameter (indicated by the black arrows), signifying increased vascular abnormalities and enhanced blood flow.

**Figure 2:**
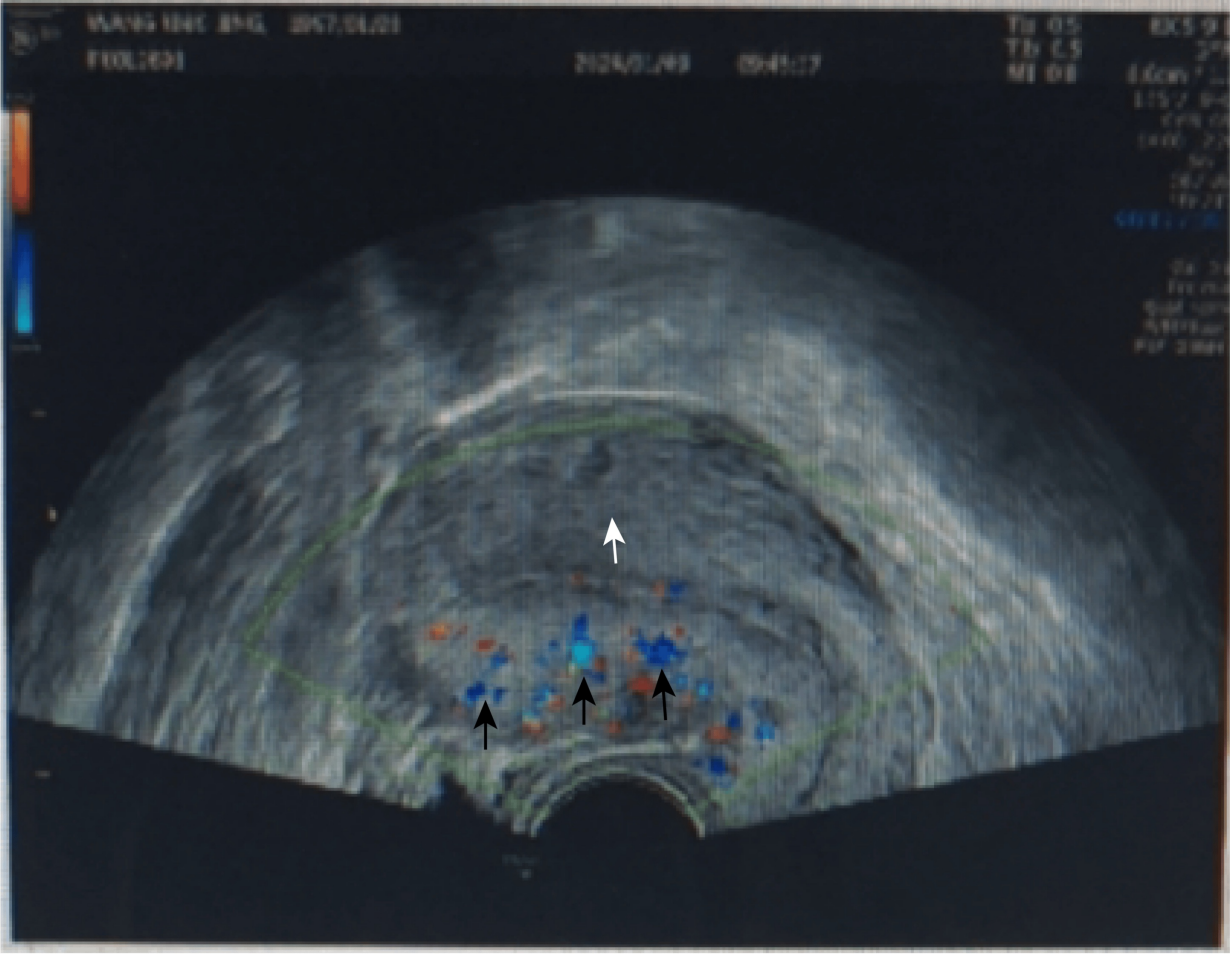
In the non-pregnant state, the anterior and posterior myometrial walls have equal thickness. The upper myometrium (indicated by the white arrow) and the underlying blood flow (indicated by the black arrows) show no significant vascular filling in the affected uterine vessels.

A 27-year-old pregnant woman, gravida 2 para 0, presented in September 2024 with KTS and uterine venous malformations, with a history of swelling and redness in her left lower limb since childhood. At the age of 17, she underwent laser treatment to remove large cutaneous hemangiomas on her abdomen, back, and left leg. She also had a history of chronic anemia secondary to recurrent hematochezia and underwent hemorrhoidectomy at the age of 20. She had previously required a blood transfusion and had no history of thrombosis. There was no family history of KTS, vascular malformations, or clotting-related diseases. One year ago, she experienced a 46-day embryonic arrest and underwent an abortion, which was associated with minimal bleeding, and a Doppler ultrasound revealed tubular echolucent spaces throughout the myometrium (Figure 1). An ultrasound one month post-procedure showed normal myometrial echogenicity (Figure 2). Chromosomal analysis of the embryo identified trisomy of chromosome 4.

**Figure 3:**
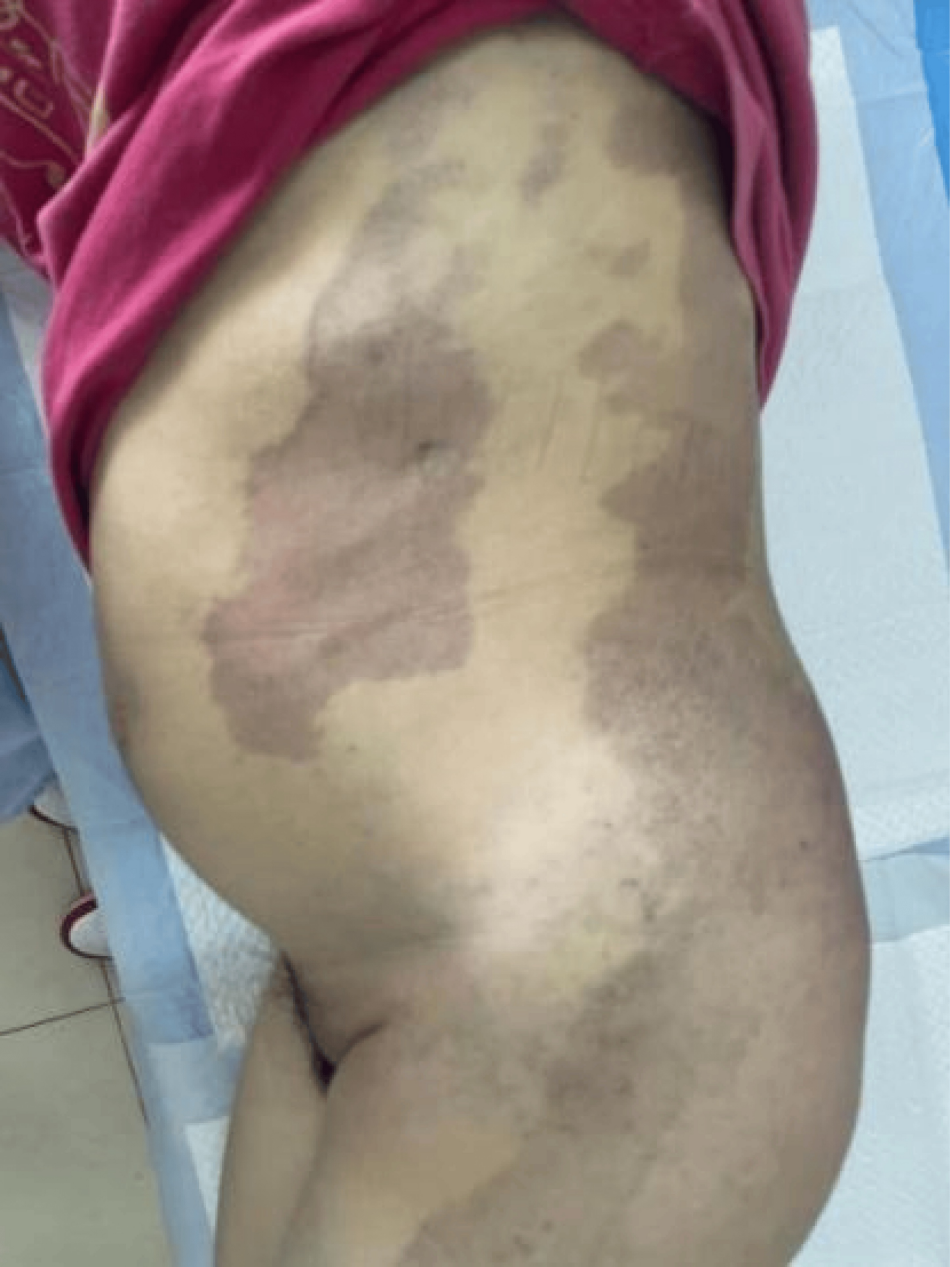
Port-wine stains (capillary malformation) on her left abdomen, buttock, leg and foot. Varicose veins in the left lower limb.

**Figure 4:**
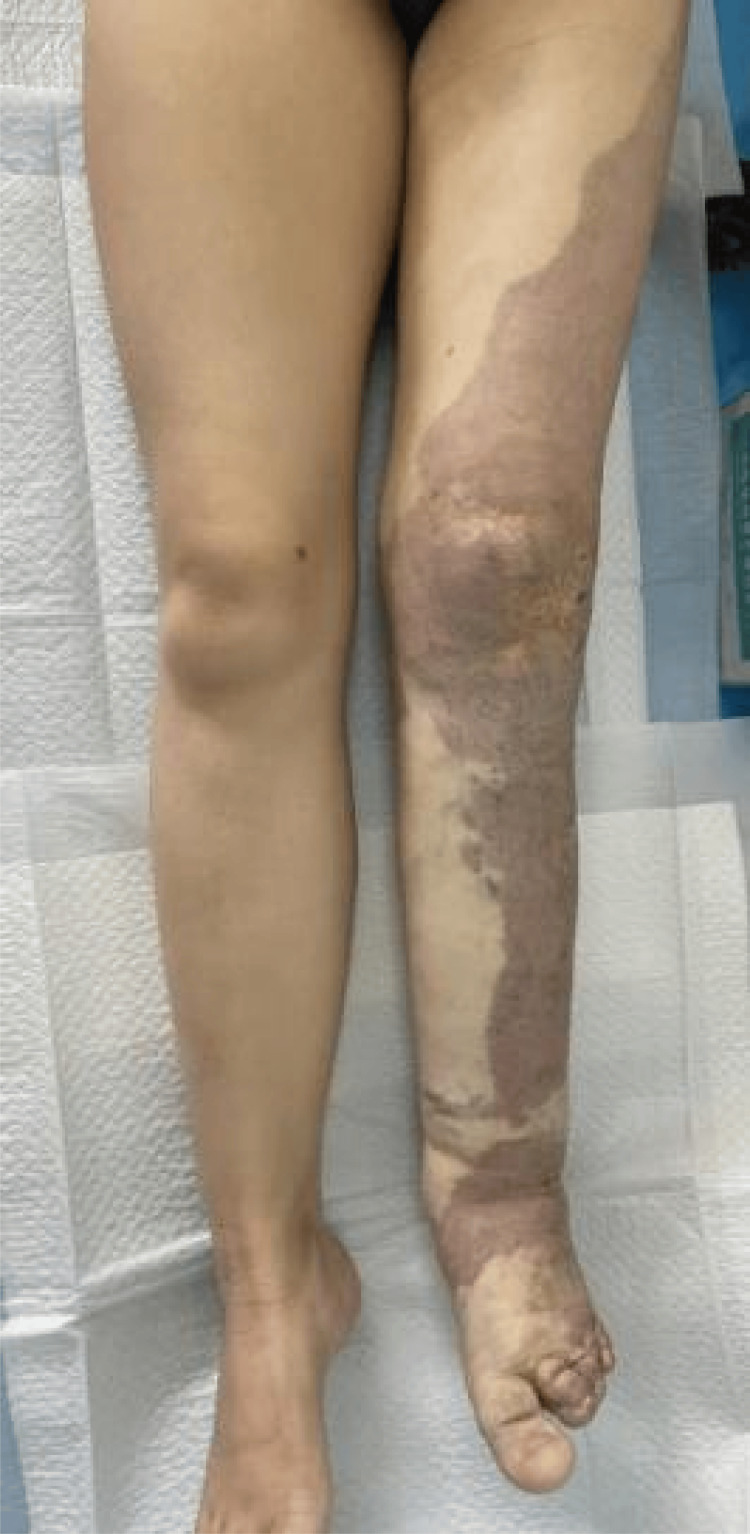
The right leg exceeded the left leg in length by 3 cm.

**Figure 5:**
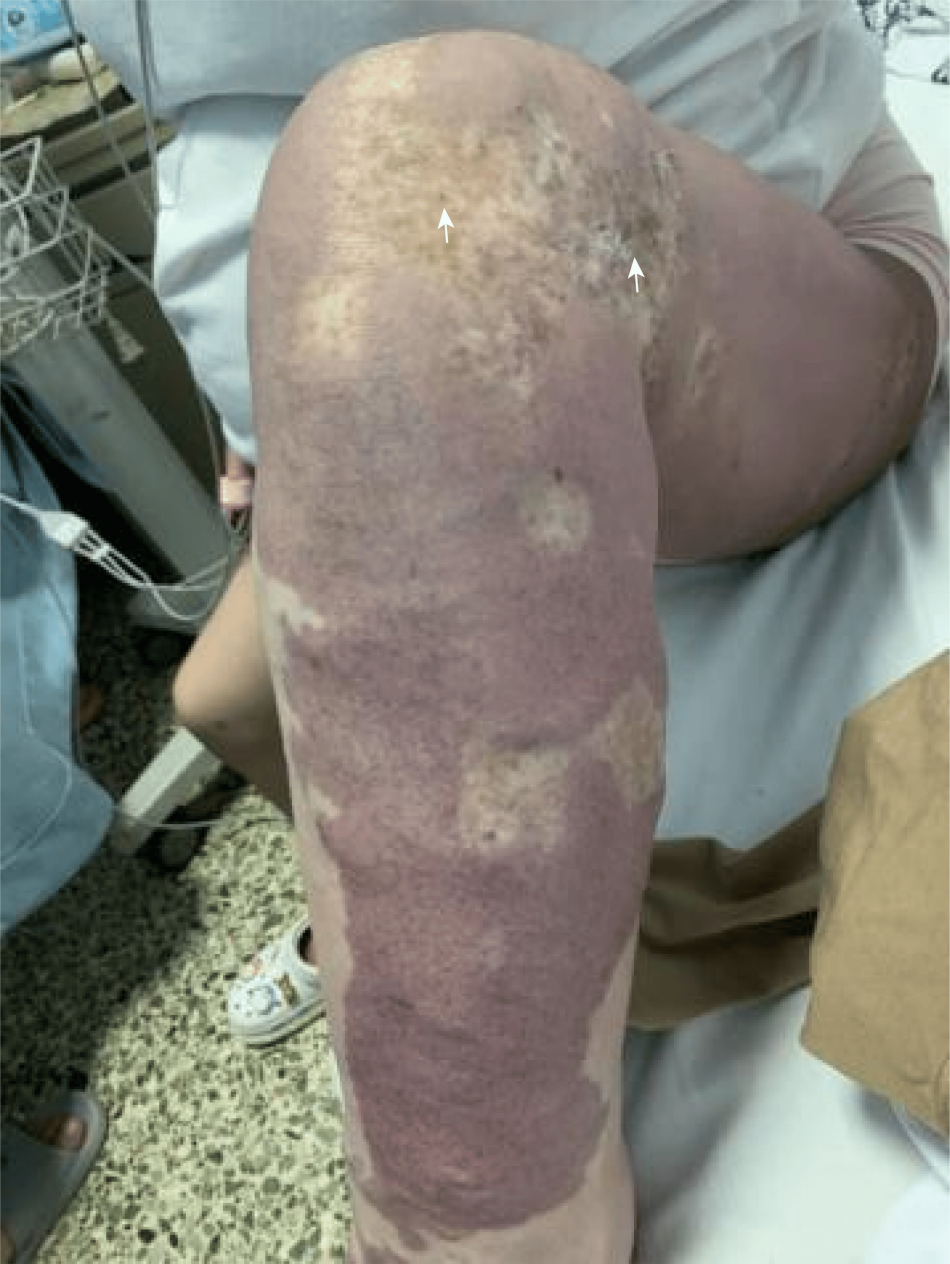
The left leg measures 2 cm more in diameter than the right at the level of patella. The left knee of the lower limb displays prominent varicose vessels (indicated by the white arrows), a site of previous laser therapy.

Her menstrual cycles were regular, occurring every 30 days with a duration of four days. Her last menstrual period was on January 29, 2024. At six weeks' gestation, Doppler ultrasound revealed diffuse tubular echolucent spaces within the myometrium. She was admitted to the hospital for severe nausea and vomiting and was treated with intravenous fluids. Additionally, she was prescribed oral progesterone for minor vaginal bleeding. Because of subclinical hypothyroidism, she was started on levothyroxine 75 μg once daily. She reported no history of fever, cold exposure, or chemical poison exposure during pregnancy. At 12 weeks' gestation, the fetal nuchal translucency measured 0.08 cm. Non-invasive prenatal DNA screening returned a low-risk result. The patient had distinctive cutaneous manifestations, including port-wine stains extending across the left leg, foot, buttocks, back, and abdomen; prominent varicose veins in the lower limb; and asymmetry in limb measurements, with her left leg being 2 cm wider at the level of the patella than the right. Additionally, her right leg was 3 cm longer than her left (Figures 3, 4, 5), confirming major diagnostic criteria of KTS, and she was referred from a primary-care hospital to the vascular surgery department. There, elevated TAT levels prompted the initiation of anticoagulation therapy. A comprehensive ultrasound assessment of uterine involvement was performed, and she was advised to wear full-length compression stockings. The patient first presented to our obstetric outpatient clinic, which has extensive experience in managing complex pregnancies, at 15 weeks' gestation.

Laboratory tests revealed white blood cell count 5.5×109/L, red blood cell count 3.04×10^12^/L, hemoglobin (HGB) 78 g/L, platelet count (PLT) 194×10^9^/L, and ferritin 6.5 ng/mL. Coagulation studies showed prothrombin time (PT) 12.3 s, activated partial thromboplastin time (APTT) 26.7 s, thrombin time (TT) 13.6 s, fibrinogen (Fib) 3.6 g/L, and D-dimer 1.97 μg/mL. Thrombosis markers indicated slightly elevated TAT at 8.23 ng/mL. A Doppler ultrasound of the bilateral lower limbs revealed no evidence of venous thrombosis. Over the previous two months, she had suffered from severe hematochezia and moderate anemia. Prior to this pregnancy, her hemoglobin level was 110 g/L. Due to persistent moderate anemia, slightly elevated D-dimer, and TAT levels, she was referred to a hematologist. She was diagnosed with iron-deficiency anemia and prescribed iron succinate supplementation. At 16 weeks' gestation, laboratory tests showed a further increase in TAT (26.79 ng/mL), plasmin-antiplasmin complex (PIC) (2.18 μg/mL), and D-dimer (3.51 μg/mL). After consulting with the hematologist, anticoagulation therapy was initiated with low molecular weight heparin (LMWH) 5000 IU subcutaneously once daily. Blood tests were conducted periodically throughout the pregnancy to monitor hemoglobin levels, platelet counts, and coagulation function (Table 1). At 20 weeks' gestation, despite continued iron supplementation and LMWH therapy, her laboratory results showed TAT 23.3 ng/mL, D-dimer 2.21 μg/mL, and hemoglobin 75 g/L.

**Table 1:** Changes in Coagulation Function, Whole Blood Routine, and Thrombosis Markers During Pregnancy and Postpartum Period PT: prothrombin time, APTT: activated partial thromboplastin time, TT: thrombin time, FDP: fibrin degradation products, Fib: fibrinogen, HGB: hemoglobin, PLT: platelet, TAT: thrombin-antithrombin III complex, PIC: plasmin-antiplasmin complex, POD: postoperative day

	PT (Normal range 9.4-12.5s)	APTT (Normal range 25.4-38.4s)	Fib (Normal range 2-4g/L)	TT (Normal range 10.3-16.6s)	FDP (Normal range 0-5.0mg/L)	Dimer (Normal range 0-0.243µg/mL)	HGB (Normal range 115-150g/L)	PLT (Normal range 125-350×109/L)	TAT (Normal range 0-4.0ng/mL)	PIC (Normal range 0-0.85µg/mL)
15 weeks	12.3	26.7	3.6	13.6	26.79	1.97	78	194	8.23	
16 weeks						3.51			26.79	2.18
20 weeks	11.4	26.6	3.35	14.5	13.21	2.21	75	189	23.3	0.79
22 weeks	11.6	26.7	3.31	14.0	10.92	1.95	83	188	10.91	0.63
24 weeks	11.5	27	3.25	15.4	7.04	0.95	90		3.76	0.58
28 weeks	11.5	28.1	3.48	14.6	4.07	0.42	115	133	5.65	0.4
34 weeks	10.9	28.8	3.9	14.4	3.73	0.35	137	136	10.42	
37 weeks	10.6	27.9	4.01	14.8	3.84	0.42	144	140	10.42	
POD 1	10.9	32.7	3.75	14.8		5.38				
POD 2						1.56	134	107		
POD 4	12.0	33	1.22	21.4	211.13	45.84	140	114	54.36	12.66
POD 8	11.5	33.5	1.73	19.2	14.46	1.72				
POD 42	12.5	33.2	1.46	16.8	52.67	5.39	129	116		
POD 53	12.4	32.9	1.51	16.5	71.85	7.9	119	109	40.5	6.4
POD 3 months	11.5	31.3	2.05	14.3	11.78	1.79	140	236	7.27	1.61

**Figure 6:**
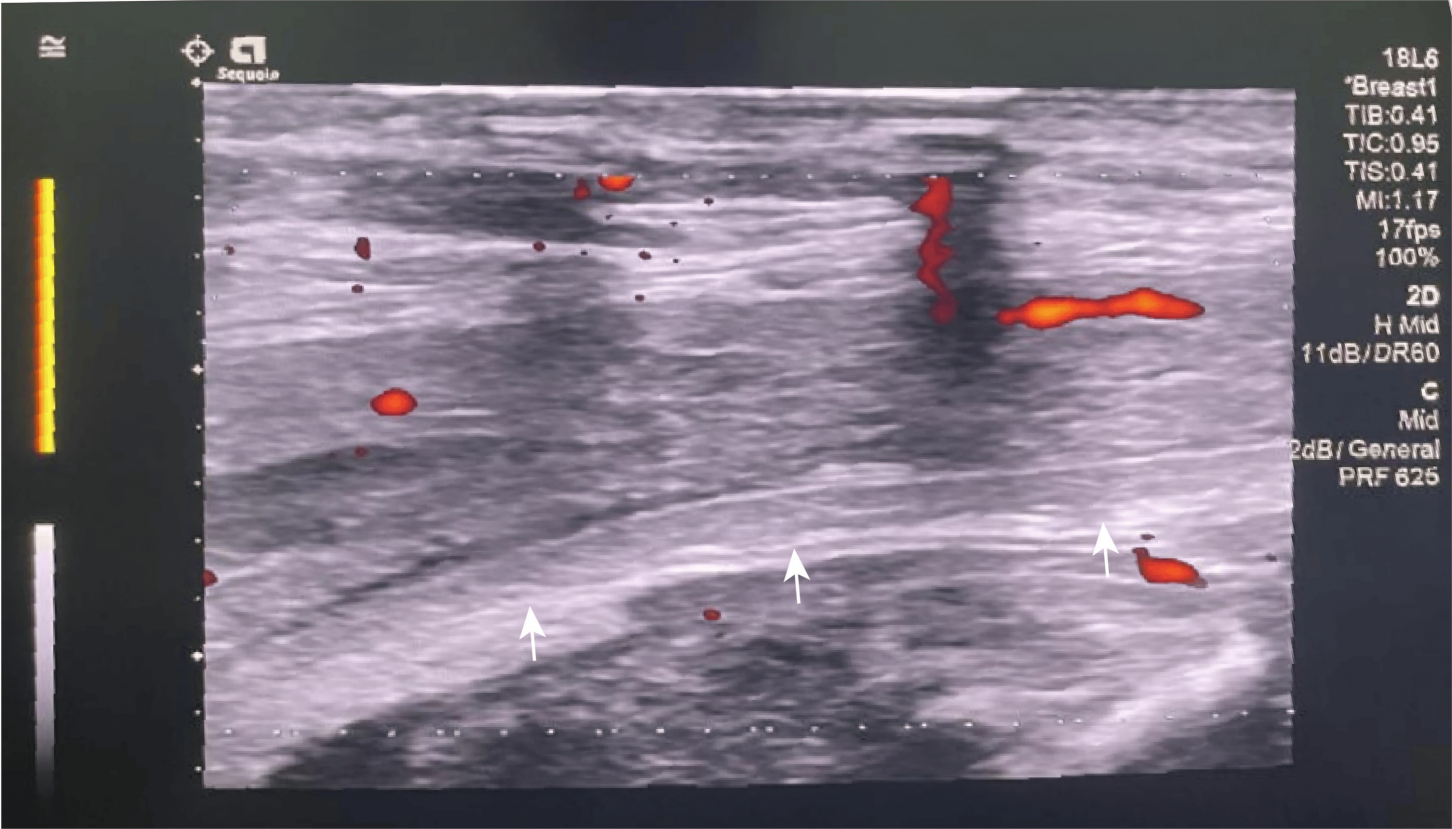
Ultrasound of the abdominal wall in the area of the cesarean section approach showed no venous malformations. The myometrium and peritoneum are not involved (indicated by the white arrows).

Serial obstetric ultrasound scans showed normal fetal growth, along with persistent thickening of the uterine myometrium with diffuse tubular echolucent spaces. Fetal echocardiography revealed no abnormalities. At 22 weeks' gestation, following six weeks of anticoagulation therapy with LMWH, TAT and D-dimer levels decreased to 10.91 ng/mL and 1.95 μg/mL, respectively. At 24 weeks' gestation, the patient was started on dietary therapy as diagnosed with gestational diabetes mellitus following an oral glucose tolerance test. At this time, TAT and D-dimer levels had normalized to 3.76 ng/mL and 0.95 μg/mL, respectively. The patient's hematochezia had resolved spontaneously. D-dimer levels remained below 0.5 μg/mL, while TAT levels fluctuated between 5.65 and 9.98 ng/mL. At 28 weeks' gestation, the hemoglobin level had increased to 115 g/L. At 34 weeks' gestation, a multidisciplinary team (MDT) comprising obstetricians, hematologists, vascular surgeons, and anesthetists convened to develop a comprehensive delivery plan. MRI imaging of the pelvis and abdomen was performed to guide anesthesia management and determine the optimal delivery method. On physical examination, her blood pressure was 120/85 mmHg, heart rate was 93 beats per minute, and her abdomen was enlarged but without visible peristaltic waves. The distribution of angiomas and varicosities remained consistent with previous findings. There was no abdominal tenderness or rebound, and the liver and spleen were not palpable. However, varicosities were palpable in both the vulva and vagina. Laboratory tests revealed white blood cell count 5.1×10^9^/L, red blood cell count 4.54×10^12^/L, HGB 137 g/L, PLT 136×10^9^/L, liver and kidney function within normal limits, and normal lipid levels. Coagulation studies showed PT 10.9 s, APTT 28.8 s, TT 14.4 s, Fib 3.9 g/L, D-dimer 0.35 μg/mL, and TAT 10.42 ng/mL. Additional tests included urine culture with no abnormalities; thyroid function tests with a thyroid-stimulating hormone (TSH) level of 5.318 mIU/L; thromboelastography (TEG) with an R time 5.9 minutes, K time 1.5 minutes, angle 69.1°, maximum amplitude 59.3 mm; autoimmune and antiphospholipid antibodies tests within normal range; and echocardiography with no structural or flow abnormalities. Colour Doppler ultrasound of the upper and lower limbs, as well as internal jugular veins, showed no evidence of venous thrombosis. Ultrasound of the abdominal wall in the planned cesarean section incision area revealed no venous malformations (Figure 6). MRI of the pelvis revealed diffuse thickening of the uterine wall and vascular malformations in the subcutaneous soft tissues of the left lumbar region, back, and buttocks.

**Figure 7:**
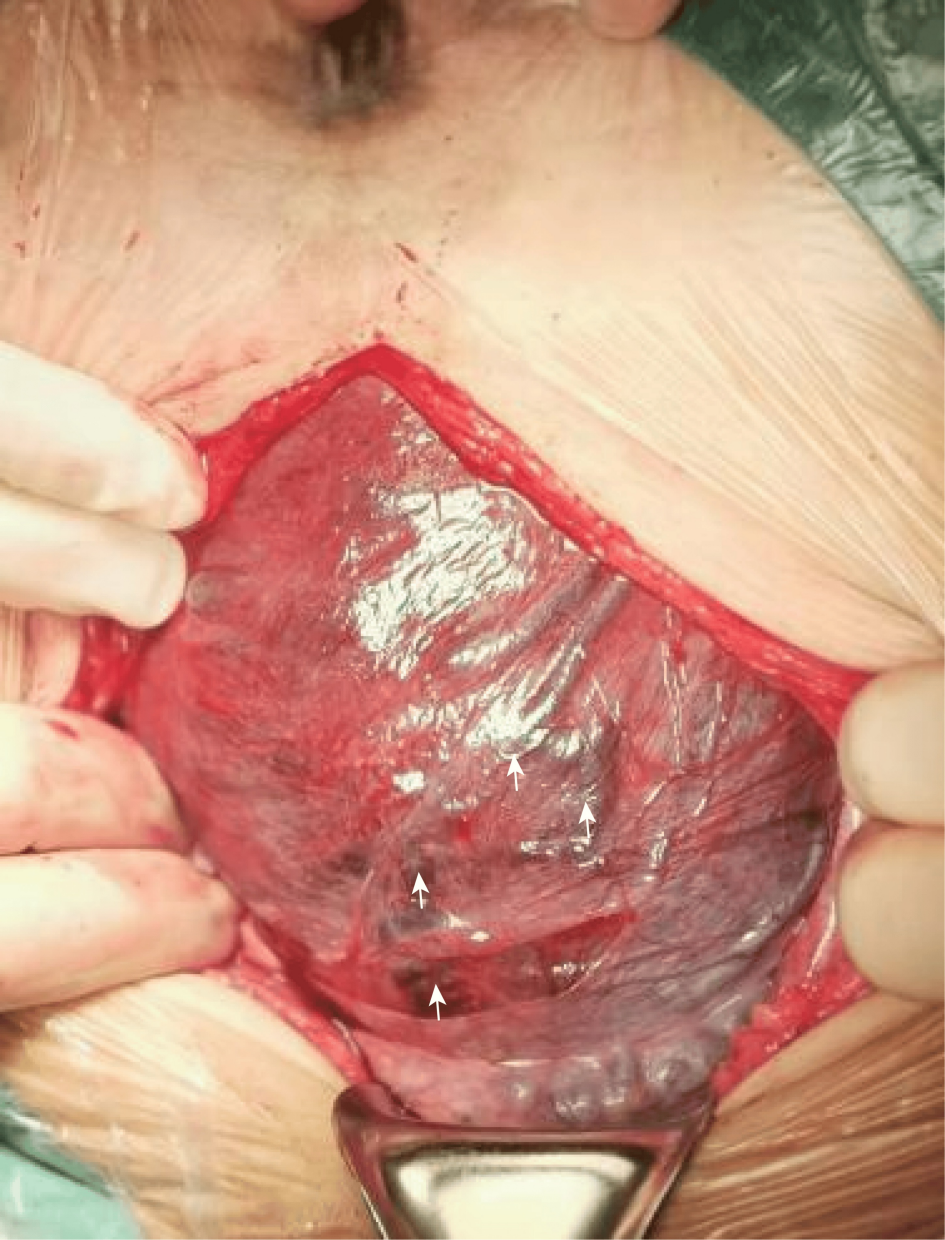
Numerous diffuse dilated vessels were present in the uterine corpus (white arrows). A venous plexus was visible on the uterovesical peritoneal fold.

**Figure 8:**
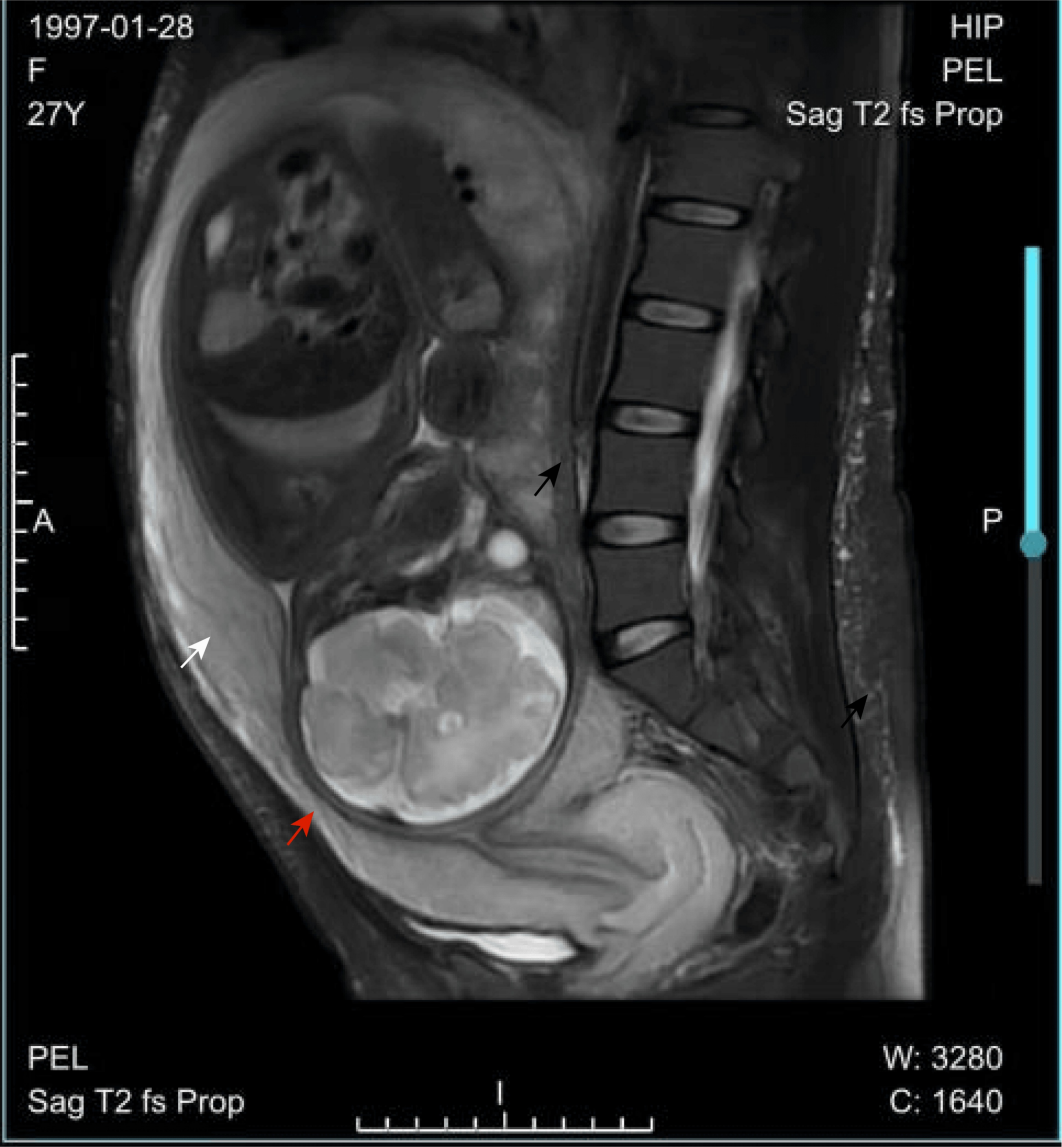
MRI of the pelvis indicated diffuse thickening of the uterine wall and the thinner lower uterine segment for the incision.

After thorough evaluation, the MDT determined that regional anesthesia was contraindicated due to the presence of extensive hemangiomas in the dorsal region. Since no esophageal or pharyngeal varicosities were detected, general anesthesia was deemed the safest approach. Given the presence of vulval and vaginal varicosities, an elective cesarean section was planned to minimize the risk of severe perinatal hemorrhage. Prenatal ultrasound revealed extensive dilation of the uterine vasculature, with no normal myometrial echogenicity observed. A scheduled cesarean section was planned, with vascular surgeons on standby to manage potential vascular complications. As the patient had a history of one embryonic demise, intends to breastfeed and to conceive again, barrier methods specifically external (male) latex condoms are recommended after puerperal state. At 37 weeks' gestation, the patient underwent a scheduled cesarean section under general anesthesia. Antenatal LMWH was temporarily discontinued 24 hours before labor. Under ultrasound guidance, a central venous catheter was placed during surgery. To avoid the hemangiomas on the left side of the abdomen, a midline vertical incision was made. No vascular malformations were observed on the abdominal wall or parietal peritoneum, however, numerous diffusely dilated vessels were present in the uterine corpus. A venous plexus was also visible on the uterovesical peritoneal fold (Figure 7). Based on prenatal MRI findings, several tortuous and dilated vessels were observed in the posterior uterine wall, and the uterine wall appeared thickened. Additionally, tortuous and dilated vessels were noted in the subcutaneous soft tissues of the left lower back and buttock (black arrow in Figure 8). The incision was made at the thinner lower uterine segment (red arrow in Figure 8). Heavy bleeding occurred at the incision site due to grossly dilated, thin-walled uterine vessels. After fetal delivery, the patient experienced profuse bleeding from uterine venous malformations. We applied local clamping, administered uterotonic agents, and ligated the ascending branches of both uterine arteries. The total surgical duration was 90 minutes, with an estimated blood loss of 2,200 mL. The patient received 500 mL of fresh frozen plasma (FFP) and approximately 700 mL of salvaged autologous red blood cells. A biopsy sample from the myometrial surgical margin was collected for gene sequencing before uterine closure. A healthy female infant weighing 2,940 g was delivered, with Apgar scores of 8/9/10. Umbilical artery blood gas analysis showed pH of 7.37, BE of -0.4 mmol/L, lactate level of 1.7 mmol/L, HGB concentration of 155 g/L, and PLT count of 237×10⁹/L. Neonatal coagulation tests and Doppler ultrasound examinations of the abdomen, heart, and brain were normal.

**Figure 9:**
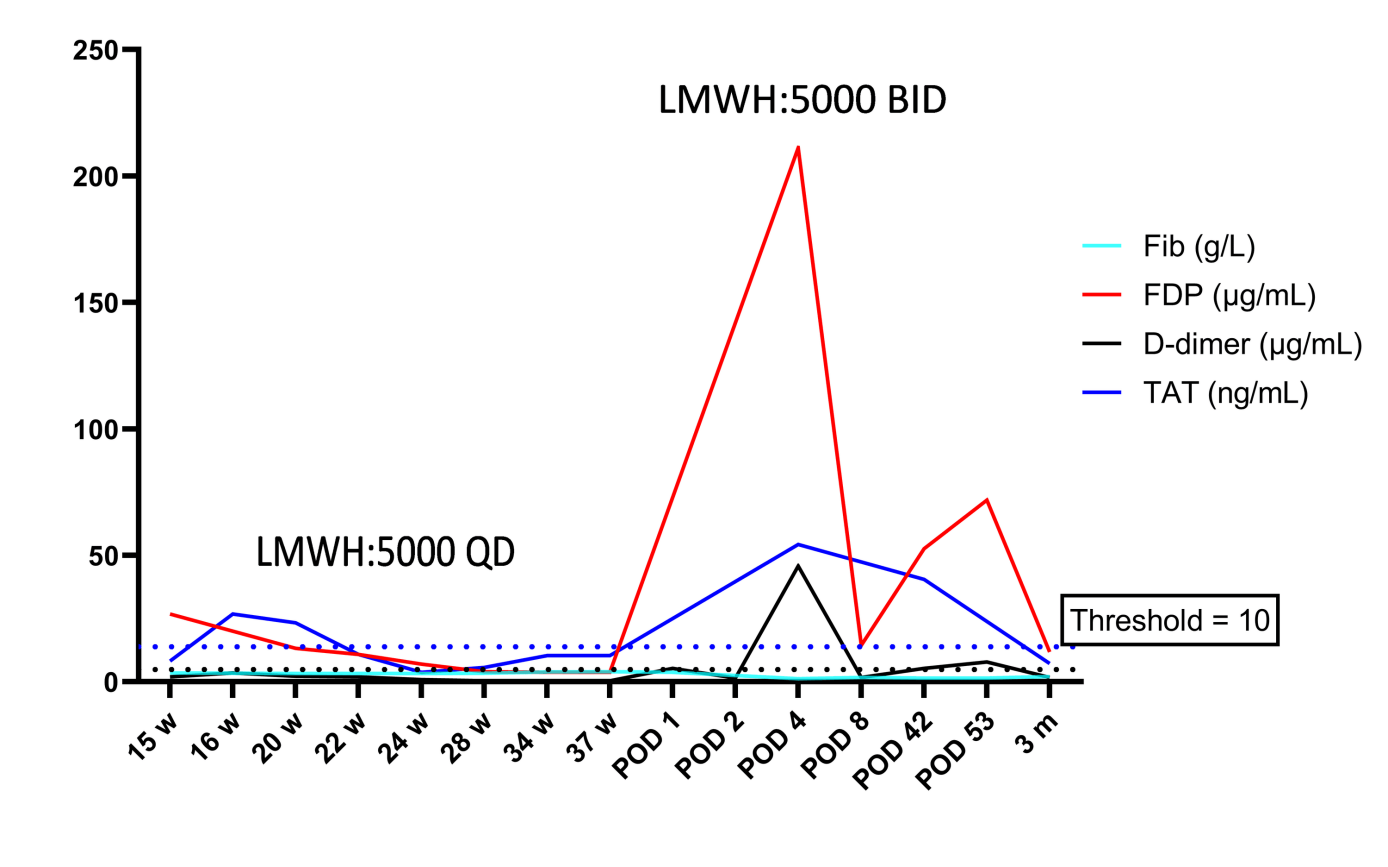
Timeline aligning key interventions with laboratory trends, showing the temporal relationship between LMWH dose adjustments (arrows) and peaks in TAT and D-dimer; the blue dashed line indicates the TAT intervention threshold and the black dashed line indicates the D-dimer intervention threshold. LMWH: low molecular weight heparin, TAT: thrombin-antithrombin III complex, FDP: fibrin degradation products, Fib: fibrinogen, POD: postoperative day

Postoperatively, LMWH 5000 IU was resumed subcutaneously once daily, and compression stockings were applied 12 hours after delivery. On postpartum day 1, coagulation function tests (PT, APTT, and TT) were within normal limits, while Fib and D-dimer levels were 3.75g/L and 5.38 μg/mL, respectively. On postpartum day 2, D-dimer, HGB, and PLT levels were 1.56 μg/mL, 134 g/L, and 107×109/L, respectively. On postpartum day 4, D-dimer and TAT levels had increased significantly to 45.84 μg/mL and 54.36 ng/mL, respectively. Laboratory tests revealed a Fib level of 1.22 g/L, TT of 21.4 seconds, and fibrin degradation products (FDP) level of 211.13 mg/L, indicating coagulation dysfunction. Blood gas analysis showed an oxygen pressure of 85 mmHg, ruling out pulmonary embolism. Doppler ultrasound of the lower limbs demonstrated dilated intermuscular veins but no evidence of deep vein thrombosis. Secondary fibrinolysis due to consumptive coagulopathy was suspected, given the presence of extensive venous anomalies. TAT is markedly elevated and fibrinogen decreases indicates a hypercoagulable state or early disseminated intravascular coagulation (DIC), LMWH inhibits thrombin-mediated endothelial injury and inflammatory response through its dual anticoagulant and anti-inflammatory effects, the LMWH dosage was increased to twice daily. The patient and infant were discharged on postpartum day 8 with a D-dimer level of 1.72 μg/mL. LMWH 5000 IU was continued subcutaneously twice daily for six weeks. At her six-week postpartum visit, the patient reported minimal vaginal bleeding and her uterus had returned to a normal size. Coagulation tests showed PT, APTT, and TT within normal limits, while Fib remained low at 1.46g/L, and D-dimer was elevated at 5.39 μg/mL. LMWH was reduced to once daily. On postpartum day 53, laboratory tests revealed Fib 1.51 g/L, D-dimer 7.9μg/mL, TAT 40.5 ng/mL, and PLT 109×10^9^/L. Kasabach-Merritt syndrome (KMS) was suspected, prompting initiation of sirolimus at 1.5 mg orally once daily. At her three-month postpartum follow-up, Fib levels had increased to 2.05 g/L, while D-dimer and TAT levels had decreased to 1.79 μg/mL and 7.27 ng/mL, respectively. During the four-month follow-up, coagulation test results returned to normal (timeline aligning key interventions with laboratory trends in Figure 9).

## Discussion

KTS is a congenital vascular malformation involving capillary, lymphatic, and venous channels. Its hallmark features predominantly affect one lower limb (85%) and rarely involve both limbs (12.5%). KTS should be distinguished from Klippel-Trènaunay-Weber syndrome (KTWS) and Parkes-Weber syndrome (PWS) which are also characterized by high-flow arteriovenous fistulae, whereas KTS is associated with low-flow venous malformations [5]. Histopathologically, KTS is marked by hyperkeratosis of the epidermis, with thin-walled, dilated, and congested blood vessels in the dermis. The deep dermis and subcutaneous tissue typically appear normal but may present as verrucous hemangiomas [6]. Venous abnormalities can also affect the oral cavity, head and neck, trunk, and buttocks, including cellulitis, headaches, cerebral hemorrhage, hydrocephalus, seizures, and visual impairment. Venous abnormalities extend to the intestinal mucosa, genitourinary tract, spleen, and abdomen, potentially leading to bowel wall deformities [7].

Although generally benign, KTS can lead to thrombotic events, visceral bleeding, and other vascular complications such as severe hematuria and hematochezia (fewer than 5%). The most serious complication of KTS is the spontaneous rupture of splenic hemangiomas [8]. This patient exhibited a limp, as well as an enlarged but shorter left leg compared to the right, accompanied by visible venous dilation and port-wine stains on the left abdomen, back, buttock, leg and foot since childhood. Based on two of the criteria, the diagnosis of KTS was clear. Seven years ago, she underwent a hemorrhoidectomy due to recurrent hematochezia and anemia, because of intestinal mucosal involvement. At 15 weeks' gestation, the patient developed moderate anemia (hemoglobin 78 g/L) due to severe hematochezia, which gradually improved after LMWH therapy at 16 weeks' gestation. Fourteen years ago, the author managed a pregnant woman with KTS complicated by peritoneal involvement. Due to inadequate preoperative assessment of venous malformations, massive bleeding ensued upon entry into the abdominal cavity, where the peritoneum was covered with numerous large, palpable angiomas. Blood loss reached 2500 mL before fetal delivery, with an additional 3500 mL of hemorrhage from the peritoneal angiomas during abdominal closure. When all conventional hemostatic measures failed, hemostasis was ultimately achieved by abdominal packing with gauze. The patient was admitted to the intensive care unit and required extensive transfusion, including 40 units of red blood cells, 3800 mL of FFP, 60 units of cryoprecipitate, and 16 units of platelets over 16 postoperative days [9]. In light of previous experiences, a thorough preoperative assessment of venous malformations via radiological imaging is crucial for determining the appropriate anesthesia and delivery approach. Accordingly, at 34 weeks' gestation, colour Doppler ultrasound and MRI of the abdomen and pelvis were performed to evaluate the extent and severity of vascular malformations involving the lumbar region, epidural and spinal spaces, airway, genital tract, uterus and lower abdominal wall. This information is essential for determining the optimal route of delivery and anesthesia options, which could result in uncontrollable hematoma formation. In some cases, general anaesthesia is preferred, as increase the risk of spinal hematoma, neurological injury, and bleeding complications during regional anesthesia. If involving the oropharyngeal region, due to the potential presence of oropharyngeal neurofibromas or soft tissue overgrowth in the palatal area, laryngoscopic assessment of the airway is necessary [10].

Delivery in KTS pregnancies requires MDT planning; a 2017 review found ~70% undergo cesarean section, driven mainly by obstetric and coexisting arteriovenous malformations [11]. In this patient, cesarean delivery under general anesthesia was preferred due to extensive varicosities involving the back and vagina, because of increased risk of rectal bleeding and haemorrhage during vaginal delivery. Compared to the four previously published cases, pregnancies with KTS that lack prenatal imaging evaluation frequently result in catastrophic bleeding, ranging from 2,000 to 6,100 mL, caused by diffuse uterine vascular malformations, necessitating hysterectomy. In contrast, our patient underwent first-trimester ultrasound and MRI at 37 weeks to delineate the extent of myometrial vascular involvement. Preoperative ultrasound revealed that the entire myometrium was occupied by extensively dilated tubular structures, with no normal myometrial echoes visible. This information guided the selection of a uterine incision that avoided the most vascular areas, pre-deposit of autologous blood, and prophylactic bilateral ascending uterine artery ligation, limiting blood loss to 2,200 mL while preserving the uterus. Postpartum low-dose, short-course anticoagulation prevented thrombosis without provoking re-bleeding, and neonatal weight and Apgar scores surpassed those in previous reports [12-14]. During surgery, the entire uterine surface was found to be covered with varicose veins. After fetal delivery, the uterine incision was swiftly clamped as there was significant bleeding due to the dilated vein, followed by the administration of oxytocin and ergometrine. Simultaneously, the ascending branches of the uterine artery were ligated, which significantly reduced blood loss. In our case, comprehensive preparations were made before laparotomy, including the administration of tranexamic acid, autotransfusion, uterine artery embolization, the presence of experienced vascular surgeons, and a contingency plan for hysterectomy if necessary. Once the uterus was sutured, vaginal bleeding became minimal. Hysterectomy remains the most common treatment for diffuse uterine venous malformations when bleeding is uncontrollable.

Imaging studies are crucial for assessing the extent of vascular malformations, particularly arteriovenous malformations. Doppler ultrasound is effective for diagnosing venous malformations, while CT scans require informed consent, due to concerns about radiation exposure and potential nephrotoxicity. MRI is the preferred imaging modality for confirming vascular lesions, as it accurately assesses the size, extension, and anatomical relationships of the malformation, and is a safer imaging option during pregnancy. Intravenous gadolinium-based contrast agents can help differentiate between venous and occult arterial malformations [13], though their use should be approached with caution during pregnancy. KTS with uterine involvement, Doppler ultrasound did not reveal any abnormalities in the uterus in the first trimester. However, color Doppler ultrasound identified tubular echolucent spaces throughout the myometrium, with detectable blood flow within some cystic lesions. MRI revealed significant uterine enlargement and diffuse myometrial thickening. Dynamic contrast-enhanced (DCE) MRI with gadolinium demonstrated slow, homogeneous enhancement of the vascularity of the myometrial lesion [13]. In this case, Doppler ultrasound was repeatedly used throughout pregnancy to monitor uterine venous malformations, while MRI was performed before delivery and was instrumental in guiding distribution of vascular malformations in the uterine muscle layer and the optimal placement of uterine incision. The imaging is also useful in identifying discrepancies in fetal lower limb length, cystic changes in the subcutaneous tissue, fetal edema, and polyhydramnios [15]. Additionally, dermoscopy can provide further diagnostic confirmation by red, dilated, linear, and arborizing blood vessels. Studies link PIK3CA mutations in pediatric KTS tissues to promising early-treatment targets [16].

Large hemangiomas and varicosities can trap significant amounts of blood, sequester platelets, and activate the coagulation cascade, which are the most commonly reported serious complications of KTS. This condition is characterized by reduced Fib levels (0.5-1 g/L), elevated D-dimer levels (2-64 μg/mL), and increased soluble FDP, while platelet counts are typically normal or mildly reduced [17]. A TAT level >10 ng/mL indicates significant coagulation system activation, which increases the risk of thrombus formation. However, TAT levels are associated with gestational age. A PIC level >2.0 μg/mL potentially leads to hyperfibrinolysis and an increased risk of bleeding. The tissue-type plasminogen activator-inhibitor complex (t-PAIC) indicates the balance between activation and inhibition of the fibrinolytic system, while thrombomodulin (TM) reflects the vascular endothelial damage. A large cross-sectional study of 97 pregnancies and 86 deliveries from 43 patients with KTS reported these risks [18]. KTS-related symptoms were aggravated during pregnancy in 43% of patients. Deep vein thrombosis (DVT) occurred in 5.8%, pulmonary embolism in 2.3%, and severe postpartum haemorrhage (PPH) in 11%, compared to 5.8%, both significantly higher than the reference population (p<0.0001). In one case of uterine involvement, a KTS patient at 39 weeks who did not receive LMWH experienced 1500 mL of bleeding during cesarean section and 4350 mL of vaginal bleeding by postpartum day 10 because of DIC necessitating a hysterectomy and abdominal gauze packing [14].

LMWH enhances antithrombin activity, reduces thrombus formation, and helps regulate the fibrinolytic system in KTS patients. In this case, at 16 weeks of gestation, TAT and D-dimer levels rose significantly, showed a significant reduction with levels remaining within the normal range until delivery after six weeks of LMWH therapy and compression stockings. Anticoagulation therapy with LMWH should begin after conception and be adjusted based on TAT and D-dimer levels, preventing false positives from an isolated increase in D-dimer. In this case, the risk of DIC increases postpartum due to rapid hormonal decline, hemodynamic shifts, and elevated inflammatory mediators such as tumor necrosis factor α (TNF-α) and interleukin-6 (IL-6), which stimulate vascular endothelial cells and platelets, enhance thrombin inhibition by antithrombin, and activate the extrinsic coagulation pathway. D-dimer and TAT levels were abnormally elevated on the fourth day postpartum. After ruling out venous thrombosis, LMWH dosage was adjusted to twice daily, and D-dimer levels returned to normal by the eighth postpartum day. In line with previous literature recommendations, we advocate the use of LMWH during the antenatal and postnatal periods to reduce thrombotic risks, as these risks outweigh the potential bleeding risks [19]. Applied compression stockings and prophylactic anticoagulation are recommended postpartum, are effective in alleviating symptoms such as limb swelling, edema, and pain, with pressure levels typically ranging from 20 to 40 mmHg [20]. Successful management of pregnancy with KTS and uterine involvement demands multidisciplinary care, involving obstetrics, anaesthesia, haematology, and vascular surgery, and vigilant thrombosis management from antepartum through postpartum.

## Conclusions

Pregnant KTS patients require compression stockings and prophylactic LMWH from early gestation through the postpartum period, with serial TAT and D-dimer monitoring to guide prompt dose adjustments. A multidisciplinary team (obstetrics, anaesthesiology, haematology, vascular surgery) oversees care, while serial prenatal ultrasound and MRI monitor disease extent and inform delivery planning.

## References

[1] Güngor Gündoğan T, Jacquemyn Y (2010). Klippel-Trenaunay syndrome and pregnancy. Obstet Gynecol Int.

[2] al-Salman MM (1997). Klippel-Trénaunay syndrome: clinical features, complications, and management. Surg Today.

[3] Luks VL, Kamitaki N, Vivero MP (2015). Lymphatic and other vascular malformative/overgrowth disorders are caused by somatic mutations in PIK3CA. J Pediatr.

[4] Canaud G, Hammill AM, Adams D, Vikkula M, Keppler-Noreuil KM (2021). A review of mechanisms of disease across PIK3CA-related disorders with vascular manifestations. Orphanet J Rare Dis.

[5] Ziyeh S, Spreer J, Rössler J, Strecker R, Hochmuth A, Schumacher M, Klisch J (2004). Parkes Weber or Klippel-Trenaunay syndrome? Non-invasive diagnosis with MR projection angiography. Eur Radiol.

[6] Supekar BB, Chopkar AD, Wankhade VH, Singh RP, Bhat DM, Suresh P (2020). Klippel-Trenaunay syndrome with arterio-veno-lymphatic malformation: a rare presentation. Indian Dermatol Online J.

[7] Li LL, Xie R, Li FQ, Huang C, Tuo BG, Wu HC (2023). Easily misdiagnosed complex Klippel-Trenaunay syndrome: a case report. World J Clin Cases.

[8] Lanjewar DN, Chothani KP, Vaishnav MV, Rao G (2024). Hemangiomatosis of the spleen in a patient with Klippel-Trenaunay syndrome: a case report. Indian J Pathol Microbiol.

[9] Jiang YP, Li QQ, Chen D (2011). Pregnancy in women with Klippel-Trenaunay syndrome: a case report and literature review. Zhonghua Fu Chan Ke Za Zhi.

[10] Sangeetha RP, Baskar N, Kamath S, Dixit P (2019). Craniotomy in Klippel-Trenaunay syndrome: concerns and challenges. Indian J Anaesth.

[11] Keepanasseril A, Keerthana K, Keepanasseril A, Maurya DK, Kadambari D, Sistla S (2017). Pregnancy in women with Klippel-Trenaunay syndrome: report of three pregnancies in a single patient and review of literature. Obstet Med.

[12] Cucinella G, Di Buono G, Geraci G (2022). Uterine involvement in Klippel-Trenaunay syndrome: a rare but relevant event. Review of the literature. Front Surg.

[13] Yara N, Masamoto H, Iraha Y (2016). Diffuse venous malformation of the uterus in a pregnant woman with Klippel-Trénaunay syndrome diagnosed by DCE-MRI. Case Rep Obstet Gynecol.

[14] Zhang J, Wang K, Mei J (2019). Late puerperal hemorrhage of a patient with Klippel-Trenaunay syndrome: a case report. Medicine (Baltimore).

[15] Orlandi G, Sarno L, Angelino A (2025). Klippel-Trénaunay-Weber syndrome: prenatal diagnosis and review of the literature. J Clin Ultrasound.

[16] Vahidnezhad H, Youssefian L, Uitto J (2016). Klippel-Trenaunay syndrome belongs to the PIK3CA-related overgrowth spectrum (PROS). Exp Dermatol.

[17] Mazoyer E, Enjolras O, Laurian C, Houdart E, Drouet L (2002). Coagulation abnormalities associated with extensive venous malformations of the limbs: differentiation from Kasabach-Merritt syndrome. Clin Lab Haematol.

[18] Horbach SE, Lokhorst MM, Oduber CE, Middeldorp S, van der Post JA, van der Horst CM (2017). Complications of pregnancy and labour in women with Klippel-Trénaunay syndrome: a nationwide cross-sectional study. BJOG.

[19] Silva Correia IF, Hussain M, Johnson JA (2025). Obstetric management for pregnant women with Klippel-Trenaunay syndrome: a UK case report and review of the literature. Int J Gynaecol Obstet.

[20] Wang SK, Drucker NA, Gupta AK, Marshalleck FE, Dalsing MC (2017). Diagnosis and management of the venous malformations of Klippel-Trénaunay syndrome. J Vasc Surg Venous Lymphat Disord.

